# Molecular docking studies for the identification of novel melatoninergic inhibitors for acetylserotonin-O-methyltransferase using different docking routines

**DOI:** 10.1186/1742-4682-10-63

**Published:** 2013-10-24

**Authors:** Syed Sikander Azam, Sumra Wajid Abbasi

**Affiliations:** 1 National Center for Bioinformatics, Quaid-i-Azam University, 45320 Islamabad, Pakistan

**Keywords:** Acetylserotonin-O-methyltransferase, Bipolar disorders, Pineal parenchymal tumors, Melatoninergic inhibitors, Molecular docking, Binding affinities

## Abstract

**Background:**

N-Acetylserotonin O-methyltransferase (ASMT) is an enzyme which by converting nor-melatonin to melatonin catalyzes the final reaction in melatonin biosynthesis in tryptophan metabolism pathway. High Expression of ASMT gene is evident in PPTs. The presence of abnormally high levels of ASMT in pineal gland could serve as an indication of the existence of pineal parenchymal tumors (PPTs) in the brain (J Neuropathol Exp Neurol 65: 675–684, 2006). Different levels of melatonin are used as a trait marker for prescribing the mood disorders e.g. Seasonal affective disorder, bipolar disorder, or major depressive disorder. In addition, melatonin levels can also be used to calculate the severity of a patient’s illness at a given point in time.

**Methods:**

Seventy three melatoninergic inhibitors were docked with acetylserotonin-O-methyltransferase in order to identify the potent inhibitor against the enzyme. The chemical nature of the protein and ligands greatly influence the performance of docking routines. Keeping this fact in view, critical evaluation of the performance of four different commonly used docking routines: AutoDock/Vina, GOLD, FlexX and FRED were performed. An evaluation criterion was based on the binding affinities/docking scores and experimental bioactivities.

**Results and conclusion:**

Results indicated that both hydrogen bonding and hydrophobic interactions contributed significantly for its ligand binding and the compound selected as potent inhibitor is having minimum binding affinity, maximum GoldScore and minimum FlexX energy. The correlation value of r^2^ = 0. 66 may be useful in the selection of correct docked complexes based on the energy without having prior knowledge of the active site. This may lead to further understanding of structures, their reliability and Biomolecular activity especially in connection with bipolar disorders.

## Background

**Figure 1:**
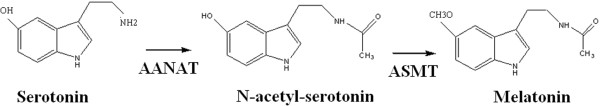
ASMT catalyzed melatonin synthesis.

N-Acetylserotonin-O-methyltransferase (ASMT) is an enzyme which by converting nor-melatonin to melatonin catalyzes the final reaction in melatonin biosynthesis in tryptophan metabolism pathway (Figure 1). The enzyme also catalyzes the conversion of 5-hydroxy-indoleacetate to 5-methoxy-indoleacetate (a second reaction in tryptophan metabolism) [1]. ASMT lies under three critically important sub-classes of enzyme, transferases, one-carbon group transferases, and methyltransferases. ASMT protein is found in both prokaryotes and eukaryotes. In humans, ASMT gene encoded this enzyme. There are two identical copies of ASMT (one on the X arm and another on the Y arm of chromosome) [2,3]. High Expression of ASMT gene is evident in pineal parenchymal tumors (PPTs). The presence of abnormally high levels of ASMT in pineal gland could serve as an indication of the existence of PPTs in the brain [4]. Different levels of melatonin are used as a trait marker for prescribing the mood disorders e.g. seasonal affective disorder, bipolar disorder, or major depressive disorder. In addition, melatonin levels can also be used to calculate the severity of a patient’s illness at a given point in time. As studies have revealed a direct correlation between the amount of ASMT in the pineal gland and the melatonin level, additional knowledge of ASMT could provide valuable insight into the nature and onset of these impairing disorders [5]. In studying the various properties associated with protein-ligand interactions, docking is a powerful tool. Since molecules in nature have a tendency to be found in their lowest energy form, the final configuration should also be of low energy [6]. Understanding these properties is crucial in the rational design of potent inhibitors.

Molecular Docking is an effective and competent tool for *in silico* screening. It is playing an important and ever increasing role in rational drug design [7,8]. Docking is a computational procedure of searching for an appropriate ligand that fits both energetically and geometrically the protein’s binding site. In other words, it is a study of how two or more molecules e.g. ligand and protein, fit together. The problem is like solving a 3D puzzle [9]. During the past decade, for understanding the formation of intermolecular complexes, the application of computational methods in this arena has been subjected to intensive research. It is commonly known that molecular binding of one molecule (the ligand) to the pocket of another molecule (the receptor), which is commonly a protein, is responsible for accurate drug activity. Molecular docking has been proved very efficient tool for novel drug discovery for targeting protein. Among different types of docking, protein-ligand docking is of special interest, because of its application in medicine industry [10]. Protein-ligand docking refers to search for the accurate ligand conformations within a targeted protein when the structure of proteins is known [11].

Docking procedures are basically the combination of search algorithms and scoring function. The largest number of search algorithms and scoring functions are available. Search algorithms predict the ligand binding orientation and conformations commonly referred to as posing [11]. Some common search algorithms are [9]: Monte Carlo methods, Genetic algorithms, Fragment-based methods, Point complementary methods, Distance geometry methods, Tabu searcher and Systematic searches. In order to differentiate between the active and random compounds, the scoring functions are employed. The scoring functions predict binding free energies in ligand-protein docking generally in 7–10 kJ/mol [12]. Numbers of molecular docking software are employed in drug research industry [9]. The most popular and commonly used softwares for molecular docking are AutoDock [13-15], AutoDock/Vina [16], GOLD [17,18], FlexX [19], FRED [20], DOCK [21] and ICM [22]. For docking purpose, AutoDock/Vina employed Broyden-Fletcher-Goldfarb-Shanno algorithm and it significantly improves the average accuracy of the binding mode predictions compared to AutoDock 4 [16]. FlexX employed an IC algorithm. IC algorithm attempts to reconstruct the bound ligand by first placing a rigid anchor in the binding site and later using a greedy algorithm to add fragments and complete the ligand structure. GOLD considers the degree of freedom in the binding site that corresponds to reorientation of hydrogen bond donor and acceptor groups. This degree of freedom represents only a very small fraction of the total conformational space that is available but should account for a significant difference in binding energy values [23].

In connection with efforts rendered in searching for novel inhibitors of ASMT, we perform a comparative docking study with four extensively used programs: AutoDock/Vina, GOLD, FlexX and FRED. The docking accuracy and scoring reliability of the selected docking approaches were evaluated by docking seventy three melatoninergic ligands with ASMT and correlating the predicted binding affinities with the experimental values.

## Methods

### ASMT and melatoninergic inhibitors

The protein used in the docking study was obtained through homology modeling by Azam et al., [24]. Dogsite web server was employed to detect the binding pocket of ASMT (Table 1) [25]. Seventy three structurally diverse ASMT inhibitors (Additional file 1) with representative good biological activity were selected from the literature [26-31]. The 2D structures of the melatoninergic inhibitors were drawn using chemical structure drawing package, ChemOffice 2004 [32]. The conformational energies of inhibitors were minimized by using UCSF Chimera [33]. The minimized structures were then subjected to docking studies.

**Table 1:** Active site residues of ASMT

**Amino acids**	**One letter code**
TRP 11, 117, 285	W
LYS 107, 223	K
TYR 108 131, 336	Y
GLY 110, 263	G
SER 113	S
CYS 116	C
THR 112, 144, 207, 336	T
LEU 142, 186, 308, 326	L
GLU 152	E
PHE 26,143, 156, 237	F
ILE 277, 310	I
ASP 238, 268, 284	D
ARG 210, 280	R
ASN 330	N
VAL 333	V
GLN 253, 334	Q
MET331	M

### Docking protocol

Molecular docking protocols are widely used for predicting the binding affinities for a number of ligands. In current work, our aim was to examine the possibility of an existing relationship between the experimental bioactivities of the inhibitors under study and the docking scores. In order to get accurate results, all the docking experiments were performed with the default parameters. The time to dock one ligand was approximately 1–2 min. Docking with AutoDock/Vina, GOLD and FRED was performed on a Linux workstation (openSUSE11.4) with an Intel Pentium D processor (3.0 GHz) and 1 GB of RAM where as FlexX was run on windows 7 equipped with an Intel® Atom™ processor (1.67 GHz) and 1GB of RAM.

### Docking using AutoDock/Vina

Intermediary steps, such as pdbqt files for protein and ligands preparation and grid box creation were completed using Graphical User Interface program AutoDock Tools (ADT). ADT assigned polar hydrogens, united atom Kollman charges, solvation parameters and fragmental volumes to the protein. AutoDock saved the prepared file in PDBQT format. AutoGrid was used for the preparation of the grid map using a grid box. The grid size was set to 60 × 60 × 60 xyz points with grid spacing of 0.375 Å and grid center was designated at dimensions (x, y, and z): -1.095, -1.554 and 3.894. A scoring grid is calculated from the ligand structure to minimize the computation time. AutoDock/Vina was employed for docking using protein and ligand information along with grid box properties in the configuration file. AutoDock/Vina employs iterated local search global optimizer [34,35]. During the docking procedure, both the protein and ligands are considered as rigid. The results less than 1.0 Å in positional root-mean-square deviation (RMSD) was clustered together and represented by the result with the most favorable free energy of binding. The pose with lowest energy of binding or binding affinity was extracted and aligned with receptor structure for further analysis.

### Docking using GOLD (Genetic Optimization for Ligand Docking)

GOLD utilizes genetic algorithm to explore the rotational flexibility of receptor hydrogens and ligand conformational flexibility [18]. In GOLD docking was carried out using the wizard with default parameters population size (100); selection- pressure (1.1); number of operations (10,000); number of islands (1); niche size (2); and operator weights for migrate (0), mutate (100), and crossover (100) were applied. The active site with a 10 Å radius sphere was defined by selecting an active site residue of protein. Default Genetic Algorithm settings were used for all calculations and a set of 10 solutions were saved for each ligand. GOLD was used by a GoldScore fitness function. GoldScore is a molecular mechanism like function and has been optimized for the calculation of binding positions of ligand. It takes into account four terms:

(1) $$\begin{array}{ll} \;\mathrm{Fitness}= & S_{\left(\mathrm{hb}\_\mathrm{ext}\right)}+1.3750*S_{\left(\mathrm{vdw}\_\mathrm{ext}\right)}+S_{\left(\mathrm{hb}\_\mathrm{int}\right)}+1.0000*S_{\left(\mathrm{int}\right)}\\ & S_{\left(\mathrm{int}\right)=}S_{\left(\mathrm{vdw}\_\mathrm{int}\right)+}S_{\left(\mathrm{tors}\right)} \end{array}$$

Where S_hb_ext_ is the protein-ligand hydrogen bonding and S_vdw_ext_ are the van der waals interactions between protein and ligand. S_hb_int_ are the intramolecular hydrophobic interactions whereas *S*_vdw_int_ is the contribution due to intramolecular strain in the ligand.

### Docking using FlexX

FlexX (which is now a part of LeadIT) is a flexible docking method that uses an Incremental Construction (IC) algorithm and a pure empirical scoring function similar to the one developed by Böhm and coworkers [36] to place ligands into the active site. IC algorithms first dissect each molecule into a set of rigid fragments according to rotatable bonds, and then incrementally assemble the fragments around the binding pocket [19]. For docking studies, the pdb files of ligands were transformed into a SYBYL mol2 file format and a ligands library was generated. A receptor description file was prepared through the FlexX graphic interface. An active site was defined by selecting the residue of the protein. The active site includes protein residues around 10 Å radius sphere centered on the center of mass of the ligand. Based on energy values, top ten ranked poses for each ligand in data set were selected for further analysis.

The free binding energy ΔG of the protein–ligand complex is given by:

(2) $$\begin{array}{ll} \;\Delta G=\Delta G_{0} & +\Delta G_{\mathrm{rot}}{\mathrm{xN}}_{\mathrm{rot}}\\ & +\Delta G_{\mathrm{hb}}\sum_{\mathrm{neutral}\;H\;\mathrm{bonds}}f\left(\Delta R,\Delta \alpha \right)\\ & +\Delta G_{\mathrm{io}}\;\Sigma_{\mathrm{ionic}\;\mathrm{int}.}\;f\;\left(\Delta R,\Delta \alpha \right)\\ & +\Delta G_{\mathrm{ar}}\Sigma_{\mathrm{aro}\;\mathrm{int}.}f\;\left(\Delta R,\Delta \alpha \right)\\ & +\Delta G_{\mathrm{lipo}}\Sigma_{\mathrm{lip}\;\mathrm{ocont}.}f*\left(\Delta R\right) \end{array}$$

Here, f (ΔR, Δα) is a scaling function penalizing deviations from the ideal geometry and N_rot_ is the number of free rotatable bonds that are immobilized in the complex. The terms Δ*G*_hb_, Δ*G*_io,_ ΔG_ar_ and ΔG_0_ are adjustable parameters. ΔG_lipo_ is lipophilic contact energy (Rarey et al., [19]).

### Docking with FRED (Fast Rigid Exhaustive Docking)

FRED uses multi-conformer docking algorithm which separately generates a set of low-energy conformers, and then do rigid docking for each conformer [37]. In order to carry out correct docking, FRED required accurately prepared receptor file as well as a ligand conformer library. The receptor file was prepared by using make-receptor file provided in FRED whereas ligand conformer library was created in Omega 2.3.2 (OpenEye Scientific Software) with default settings. The volume of the docking box centered on the receptor was expanded in all directions until it was approximately 31671 Å^3^. The dimensions of the box were: 28.10 Å × 32.91 Å × 34.25 Å. FRED with a Gaussian type fitting scoring function Chemgauss4 was used to dock ASMT with ligands conformer library in order to obtain a potent inhibitor against ASMT. Chemgauss4 uses the potentials between the chemically matched positions around the ligand docked pose. Those chemical positions are complementary to the nearby specific groups in the receptor. Generally, the interactions are either hydrogen bond donors or acceptors and a favorable hydrogen bond score is obtained when a polar hydrogen position on one molecule overlaps a lone pair position on another molecule. The interactions which can be scored by Chemgauss functions are: steric, acceptor, donors, coordinating groups, metals, lone pairs, polar hydrogens and chelator coordinating groups [38].

## Results and discussion

**Figure 2:**
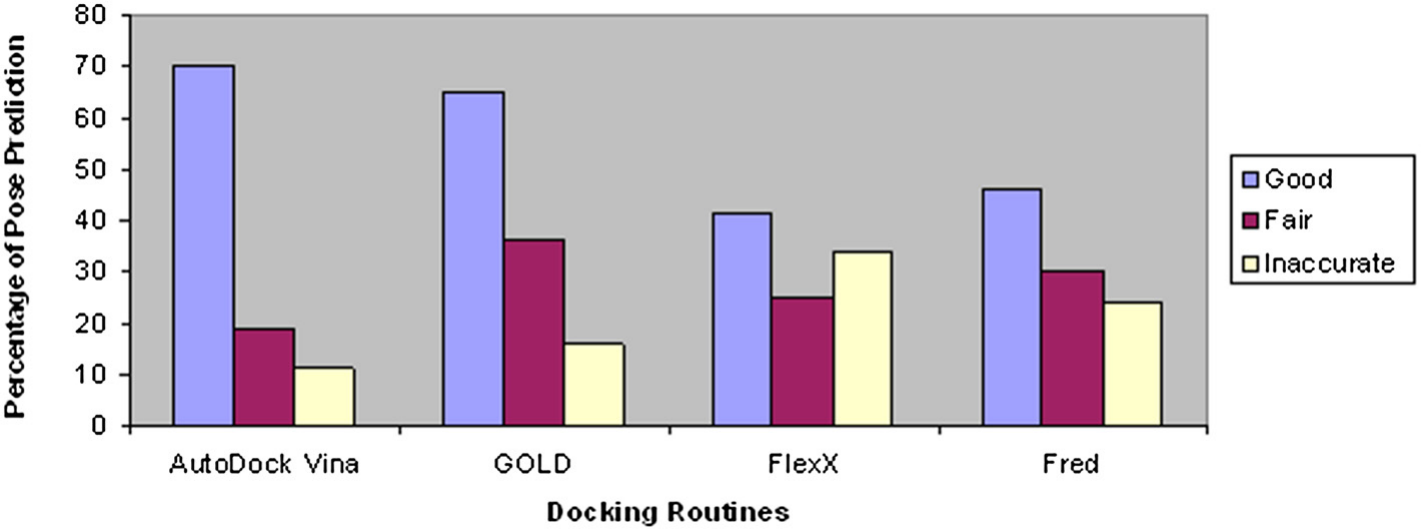
Percentages of docked poses for ASMT docked complexes obtained by the different docking routines.

In order to recognize an accurate docking routine for carrying out molecular docking studies of ASMT protein as well as to identify the potent inhibitors against that protein, four widely used docking routines (AutoDock/Vina, GOLD, FlexX and FRED) are compared in this work. Each docking routine returned top ten ranked docked poses for each ligand. The number and categories in which the dock poses fall are summarized in Table 2. Among all the studied docking routines, AutoDock/Vina was found to be the best for carrying out blind docking and in generating poses that bind best deep inside the 5 Å of the binding pocket. It generated 70% good poses. Gold and FRED also performed well with 65% and 45% of good poses respectively. FlexX returned no significant results in generating accurate poses. This variation might be because of the algorithms employed by the routines, grid box specification and active site residue specification. Overall results showed relatively poor performance ranging from 41 to 70%. These percentages were surprisingly low, indicating the docking programs often failed to find the correct binding mode. The percentages of docked poses for ASMT melatoninergic inhibitors docked complexes obtained by the different docking routines are shown in Figure 2.

**Table 2:** Number of good, fair and poor ASMT docked complexes obtained by the different docking routines

**Pose**	**AutoDock/Vina**	**GOLD**	**FlexX**	**FRED**
Good^a^	43	35	18	25
Fair^b^	18	*22*	28	13
Poor^c^	12	16	22	15

^a^good on the bases of lowest binding affinities, RMSD < 2 Å and high docked scores.

^b^fair pose on the bases of lowest to moderate binding affinities, RMSD < 3 Å and >2 Å and moderate docking scores.

^c^poor on the bases of high binding affinities, greater RMSD and lowest docked scores.

Evaluation of docking accuracy of docking programs requires the programs run at approximately comparable speeds. The average time required for carrying out the docking calculation using different scoring functions and docking routines of a single ligand is shown in Table 3. According to Table 3 in the context of the average time required for docking a single ligand, FRED is the fastest algorithm with average 1.4 sec, followed by Gold and AutoDock/Vina with an average time of 1.66 and 2–3 sec respectively. FlexX seemed to be the more time-consuming approach.

**Table 3:** Time required for the docking of a single ligand

**Docking routine**	**Docking time**
AutoDock/Vina	2-3 sec
GOLD	1.66 sec
FlexX	5 sec
FRED	1.4 sec

**Figure 3:**
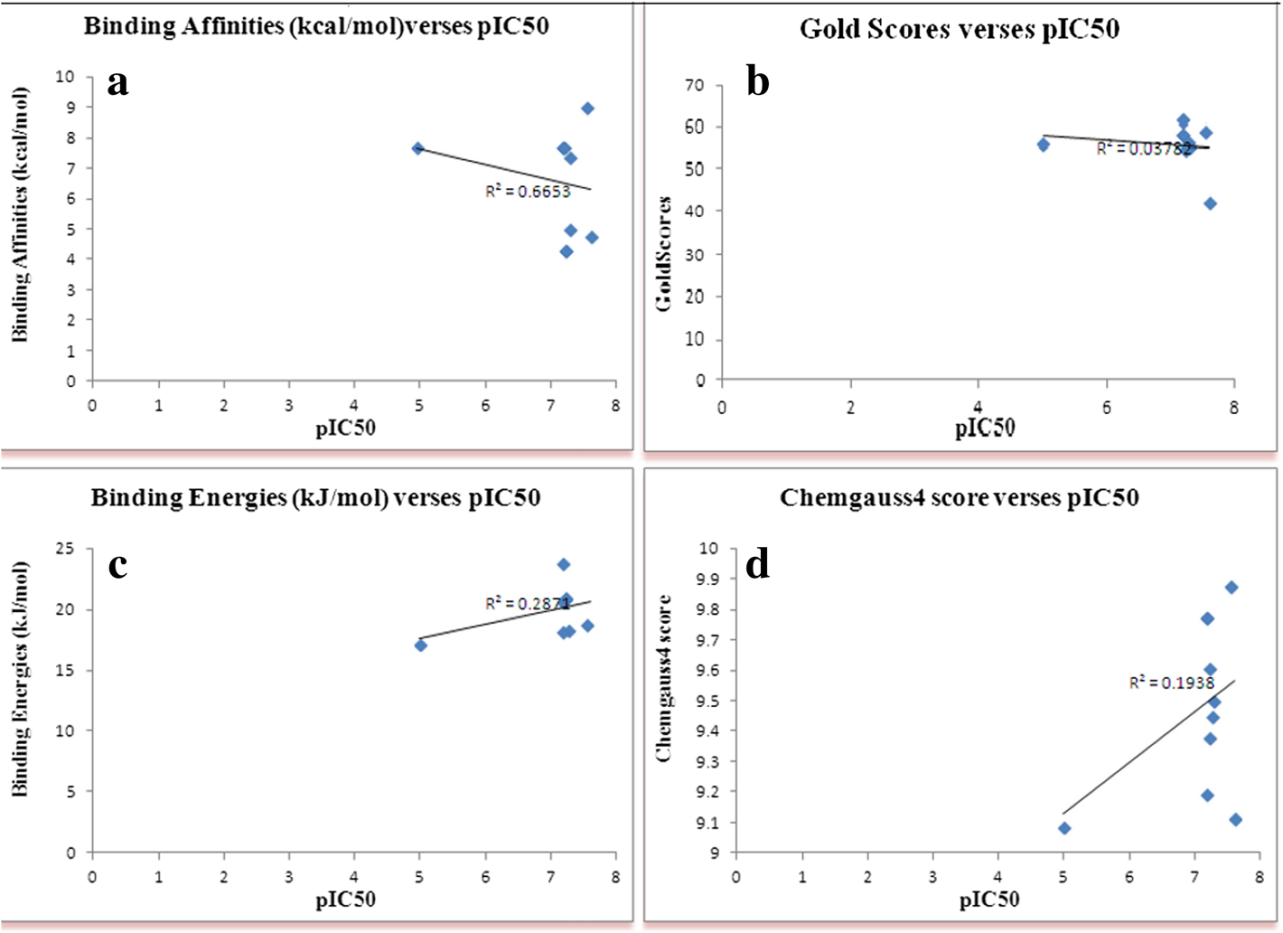
Plot showing the correlation between the experimental bioactivities (PIC50) and a) binding affinities b) GOLD scores c) Chemgauss4 scores and d) binding energies.

The correlation between the experimental bioactivities (PIC50**)** and binding affinities and the calculated scores generated by each docking routine for ASMT docked complexes are shown in Figure 3. The best squared correlation coefficient of r^2^ = 0. 66 was observed between binding affinities (AutoDock/Vina) and experimental values (Figure 3a). Low values of correlation for the Gold scores (r^2^ = 0.37) and FlexX scores (r^2^ = 0. 28) was obtained. FRED (Chemgauss4), due to its rigid-body approach, presented the lowest value of the squared correlation coefficient among the tested routines (Figure 3d).

**Figure 4:**
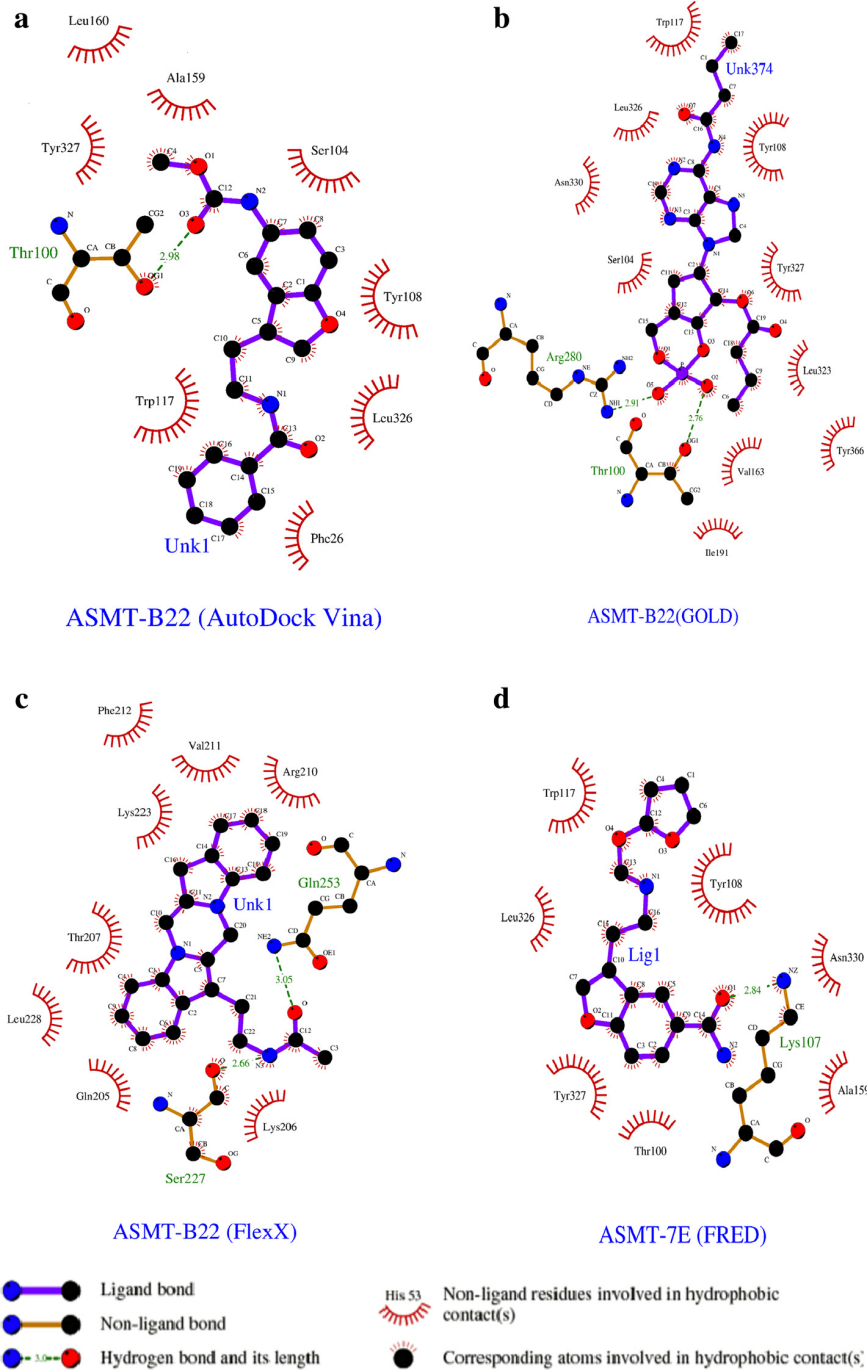
Docked conformation of ASMT with top ranked ligands showing the interaction with the crucial residues in the active site cleft using: (a) AutoDock/Vina (b) GOLD (c) FlexX (d) FRED.

**Figure 5:**
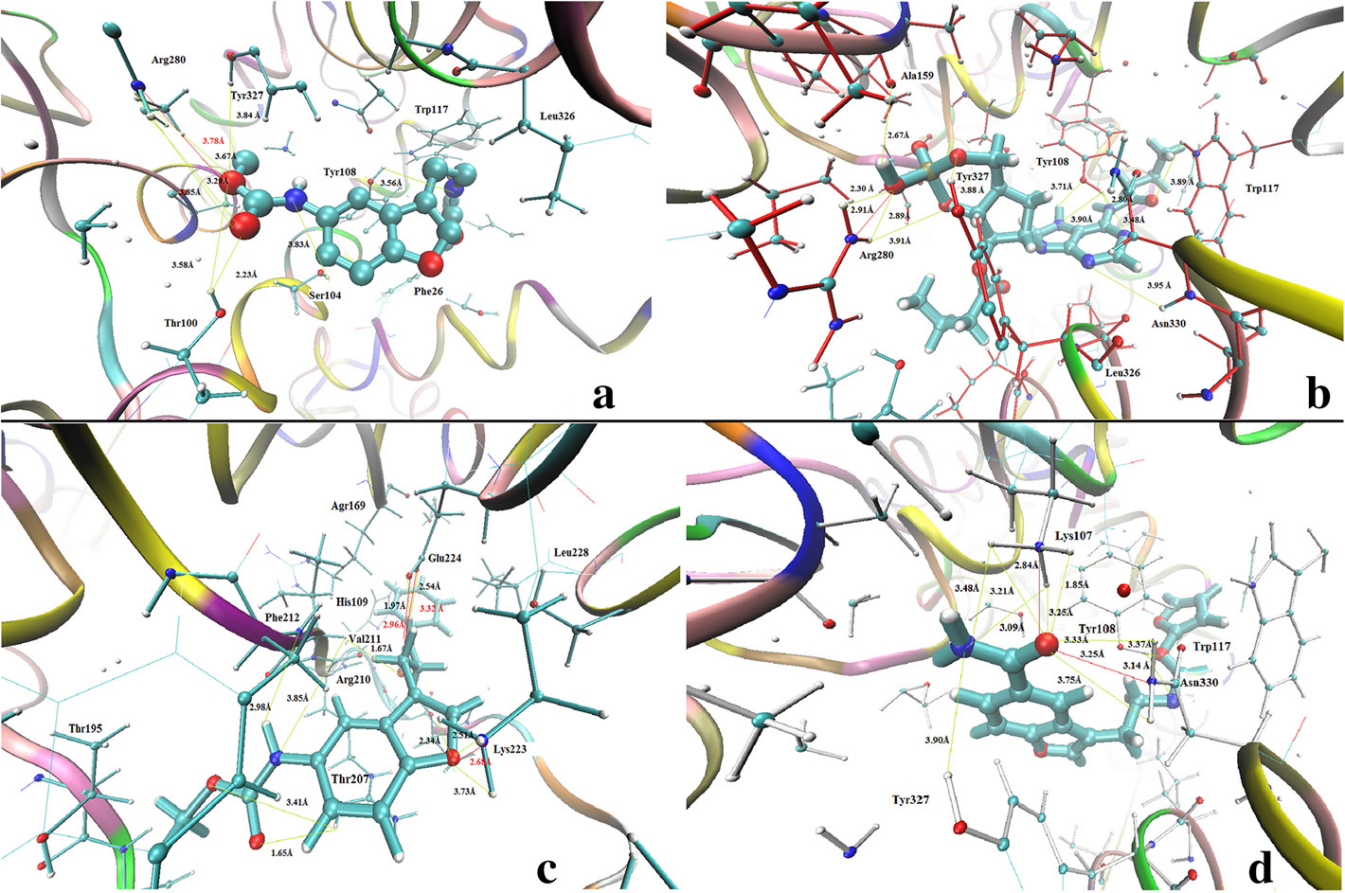
**Docked poses: Binding mode of top ranked docked poses into ASMT binding cavity: (a) AutoDock/Vina (b) GOLD (c) FlexX (d) FRED For clarity, only interacting important residues are displayed in CPK style.** The inhibitors were designed in licorice style, and part of the enzyme in the background was visualized in New Ribbon style using the VMD (Visual Molecular Dynamics) program.

The 2D view of protein–ligand interactions of the best poses generated by all the four studied routines are shown in Figure 4. As clearly depicted in Figure all molecules exhibit the same binding mode. Interestingly, important interactions can be found between these atoms and the residues Ser213, Ser98, Val97, Thr100, Val211, Ser227, Arg210, Arg280, Phe212, Leu198, Ile198, Ser104, Thr195, Leu160, Tyr327, Tyr108, Trp117, Leu326, Phe29, Phe19, Gln334, Asn330, Ala159, Lys107, Met105 which directly participate in the catalytic mechanism of this enzyme. The ligand–enzyme complex is stabilized mainly by hydrogen bonds and hydrophobic interactions. All the top docked poses generated by each docking routine exhibited well-established bonds with one or more amino acids in the binding pocket of ASMT. The top-ranked pose with lowest docked binding affinities and high docking scores are generally used as a standard selection in most of the docking programs. The best poses of ASMT-B22 generated by AutoDock/Vina, Gold and FlexX and ASMT-A3 by FRED are shown in Figure 5. For B22 the binding affinity is found to be -9.2 Kcal/mol. The orientation and hydrogen bonding, ionic interactions of B22 within ASMT active site are shown in Figure 5a.

Different sets of hydrogen bonding interactions with polar side chain residues of Arg280, Ser104, Thr100, Tyr108 and with phenol of Tyr327 are observed at distances within 4 Å. An ionic interaction with the side chain residue of Arg280 is also observed. The indole ring and other side chain carbons of B22 formed strong hydrophobic interactions with nonpolar residues Leu160, Tyr327, Tyr108, Leu326, Trp117 and Phe26 and are suggested to increase binding affinity (Table 4).

**Table 4:** Important interactions between the active residues of ASMT and Ligands within 5 Å

**Compound**	**Hydrogen bonding**		**Ionic interactions**		**Hydrophobic interactions**	
ASMTB22 (AutoDock/Vina)	Arg280:HH11…O	3.67 Å	Arg280:NH1…O	3.78 Å	Leu160:CD2…C	3.69 Å
	Arg280:HH12…O	3.29 Å			Tyr327:CE1…C	3.40 Å
	Arg280:HH12…O	3.85 Å			Tyr108:CZ…C	3.97 Å
	Ser104:HG…N	3.83 Å			Tyr108:CE2…C	3.64 Å
	Thr100:HG1…O	2.23 Å			Tyr108:CE2…C	3.77 Å
	Thr100:HG1…O	3.58 Å			Tyr108:CE2…C	3.87 Å
	Tyr108:HH…N	3.56 Å			Tyr108:CD2…C	3.92 Å
	Tyr327: HH…O	3.84 Å			Tyr108:CD2…C	3.97 Å
					Leu326:CD2…C	3.49 Å
					Leu326:CB…C	3.65 Å
					Leu326:CB…C	3.78 Å
					Leu326:CG…C	3.98 Å
					Leu326:CD2…C	3.49 Å
					Leu326:C…C	3.89 Å
					Trp117:CD1…C	3.90 Å
					Trp117:CE2…C	3.58 Å
					Trp117:CZ2…C	3.96 Å
					Trp117:CD2…C	3.62 Å
					Trp117:CD2…C	3.94 Å
					Trp117:CG…C	3.72 Å
					Trp117:CD2…C	3.73 Å
					Phe26:CZ…C	3.42 Å
					Phe:26:CE2…C	3.81 Å
GOLD	Arg280:HH12…O	2.30 Å	Arg280:NH1…O	2.91 Å	Tyr108: CE1…C	3.45 Å
	Arg280:HH11…O	2.89 Å			Tyr108: CE1…C	3.96 Å
	Arg280:HH11…O	3.91 Å			Tyr108:CE1…C	3.92 Å
	Asn330:H…N	3.95Å			Tyr108: CZ…C	3.43 Å
	Ala159:O…H	2.67Å			Tyr108: CD2…C	3.86 Å
	Tyr327:HH…O	3.88 Å			Tyr108:CD2…C	3.78 Å
	Tyr108:HH…N	3.90 Å			Tyr108:CE2…C	3.77 Å
	Tyr108:HH…N	3.48 Å			Tyr108:CZ…C	3.91 Å
	Tyr108:OH…H	3.71 Å			Tyr108:CZ…C	3.79 Å
	Ala159:O…H	2.67Å			Tyr108:CD2…C	3.87 Å
	Tyr108:HH…N	2.80 Å			Tyr327:CD1…C	3.76 Å
	Trp117:HE1…O	3.89 Å			Tyr327:CD1…C	3.74 Å
					Tyr327:CD1…C	3.58 Å
					Tyr327:CB…C	3.82 Å
					Tyr366:CE2…C	3.86 Å
					Tyr366:CE2…C	3.89 Å
					Trp117:CG…C	3.57 Å
					Trp117:CE2…C	3.98 Å
					Trp117:CZ2…C	3.51 Å
					Trp117:CH2…C	3.48 Å
					Trp117:CB2…C	3.98 Å
					Asn330:CB…C	3.53 Å
					Leu326:C…C	3.55 Å
					Leu326:CB…C	3.99 Å
FlexX	Arg210:H…O	2.22Å	Arg210:N…O	3.17Å	Thr195:CB…C	3.07 Å
	Arg210:H…N	3.32Å	Arg210:NH1…O	3.34 Å	Thr195:CG2…C	3.54 Å
	Arg210:HH11…N	3.29 Å	Lys223:NZ…O	2.68 Å	Lys223:CE…C	3.39 Å
	Arg210:HH12…O	2.69 Å	Glu224:OE2…N	2.96 Å	Lys223:CE…C	3.99 Å
	Arg210:HH11…O	3.26 Å	Glu224:OE1…N	3.32 Å	Leu228:CD2…C	3.14 Å
	Lys223:HZ3…O	2.34 Å			Leu228:CD2…C	3.51 Å
	Lys223:HZ1…O	2.51 Å			Leu228:CD1…C	3.95 Å
	Lys223:HZ2…O	3.73 Å			Arg169:CG…C	3.55 Å
	Thr207:HG1…O	1.65 Å			Arg169:CB…C	3.83 Å
	Thr207:HG1…O	3.41 Å			His209:CD2…C	2.63 Å
	Val211:O…H	1.67 Å			His209:CD2…C	3.31 Å
	Val211:N…H	3.85 Å			His209:CD2…C	3.39 Å
	Phe212:N…H	2.98Å			His209:CG…C	3.50 Å
	Glu224:OE2…H	1.97 Å			His209:CG…C	3.84 Å
	Glu224:OE1…H	2.54 Å			His209:CA…C	3.35 Å
					His209:CA…C	3.92 Å
					S His209:CB…C	3.93 Å
					Val211:CA…C	3.49 Å
					Val211:CA…C	3.51 Å
					Val211:C…C	3.04Å
					Val211:C…C	3.61Å
					Arg210:C…C	3.94 Å
					Arg210:C…C	3.96 Å
					Phe212:CB…C	3.65 Å
					Phe212:CB…C	3.62 Å
					Phe212:CD2…C	3.66 Å
					Phe212:CD2…C	3.38 Å
					Thr195:CG2…C	3.54 Å
					Thr195:CB…C	3.07 Å
ASMT-A3 FRED	Tyr108:HH…N	3.14 Å	Asn330:ND2…O	3.25 Å	Tyr327:CD1…C	3.88 Å
	Tyr108:HH…O	3.37 Å	Lys107:NZ…O	2.84 Å	Tyr327:CD1…C	3.84 Å
	Asn330:HD21…O	3.75 Å			Tyr327:CD1…C	3.61 Å
	Asn330:HD22…O	3.33 Å			Tyr327:CD1…C	3.90 Å
	Lys107:HZ3…O	1.85 Å			Tyr327:CE1…C	3.87 Å
	Lys107:HZ2…N	3.48 Å			Tyr327:CE1…C	3.80 Å
	Lys107:HZ3…N	3.09 Å			Tyr327:CE1…C	3.51 Å
	Lys107:HZ2…O	3.21 Å			Tyr327:CD1…C	3.59 Å
	Lys107:HZ1…O	3.25 Å			Tyr327:CE1…C	3.68 Å
	Tyr327:HH…N	3.90 Å			Tyr108:CZ…C	3.93 Å
					Tyr108:CE2…C	3.35 Å
					Tyr108:CE2…C	3.76 Å
					Tyr108:CE2…C	3.90 Å
					Tyr108:CD2…C	3.73 Å
					Tyr108:CZ…C	3.86 Å
					Trp117:CE2…C	3.69 Å
					Trp117:CZ2…C	3.34 Å
					Trp117:CD1…C	3.73 Å
					Trp117:CH2…C	3.45 Å

Figure 5b shows the binding mode of top pose B22ASMT complex generated by GOLD with GoldScore of 64.88. Compound B22 was mediating hydrogen bond interactions with the side chain residues of Arg280, Ala159, Tyr327 and Tyr108. The phenol and indole ring of B22 formed favorable hydrophobic contacts with Tyr108, Tyr327, Tyr366, Trp117 and Leu326 and ionic interaction with Arg280.

The FlexX generated 10 solutions for B22. The highest-ranking solution has a binding energy of -25.45 kJ/mol. Hydrogen bonds with a backbone and side chain residues of Arg210, Lys223 and Thr207, back bone residues of Val211, back bone residues of Phe212 and Glu224 are observed. The strong hydrophobic interactions were with Thr195, Lys223, Leu228, Arg169, pyrrole ring of His209, Val211 and aromatic ring of Phe212. Ionic interactions were with Arg210 and Glu224 (Figure 5c).

The top ranked pose of ASMT-A3 generated by FRED has Chemgauss4 score of -9.87. Phenolic side chain of Tyr327, Tyr108, amide side chain of Asn330 and side chain residues of Lys107 formed strong hydrogen bonding interactions with electronegative atoms of ligand. Phenol group of Tyr108, and the aromatic ring of Trp117 and Tyr 327 were highlighted as major contributor of hydrophobic interactions. A3 also resulted in favorable ionic interactions through the active site with the back bone residues of Asn 330 and side chain residues of Lys107 (Figure 5d) (Table 4). The lowest binding affinity, high GoldScore, a low binding energy and number of observed hydrogen bonding interactions between the backbone residues and the various important residues at the entrance of the pocket enables B22 to be a strongly binding inhibitor of ASMT.

As shown in Table 5, among the provided data set AutoDock/Vina docked all the seventy three inhibitors with binding affinities in a convincing range. Like AutoDock/Vina, GOLD also docked all the provided inhibitors however GOLD tends to be limited in terms of ligand flexibility. Kellenberger reported that out of 100 protein-ligand complexes, GOLD successfully docked 80% of the ligands at an RMSD value of 2.0 Å [37]. Likewise Perole [39] while doing virtual screening of HIV-1 protease established that when looking at only the top 10% of the rankings, GOLD will accurately bind 60% of the ligands. Additionally GOLD depends more on the binding site and it generates worst results when hydrophobic interactions plays an important role in binding [39].

**Table 5:** Comparison: showing the PIC50 values, chemgauss scores, binding affinities, gold scores and binding energies

**S. #**	**Ligand**	**PIC50**	**Chemgauss4 score**	**Binding affinities (kcal/mol)**	**GOLD scores**	**Binding energies (kJ/mol)**
1	**A3 Fred Best**	7.54	**-9.873667**	**-9**	59.09	-18.82
2	7e	7.17	**-9.772967**	-7.7	58.27	-20.66
3	B20	7.17	**-9.772967**	-7.7	58.31	**-23.75**
4	A7	7.21	**-9.606176**	-4.3	55.01	-20.91
5	A19	7.28	**-9.497549**	-5	55.39	ND
6	6e	7.27	**-9.448377**	-7.4	56.43	-18.29
7	A2	7.21	**-9.376864**	-4.3	54.47	-21.03
8	6b	7.18	**-9.190106**	-7.7	**61.41**	-18.23
9	B23	7.6	**-9.114123**	-4.8	42.07	ND
10	10f	4.95	**-9.08652**	-7.7	56	-17.21
11	**B22ADV,Fl.G.Best**	7.17	**-8.99517**	**-9.2**	**64.88**	**-25.45**
12	A6	8.11	-8.828648	-7.7	53.85	-17.84
13	6a	7.85	-8.7937	-7.2	55.17	-20.74
14	A4	7.15	-8.793257	-7.5	39.34	-11.62
15	7a	7.08	-8.77433	-7.2	55.26	-21.36
16	11 l	4.92	-8.765322	-4.9	46.14	**-22.29**
17	11j	4.17	-8.731167	-4.4	43.8	-18.52
18	11o	4.01	-8.722329	-4.4	40.92	-15.44
19	11 h	4.48	-8.703998	-4.7	44.39	-17.31
20	21	8.73	-8.70327	**-8.4**	57.6	-22
21	10b	4.63	-8.558372	-7.8	59.67	**-23.03**
22	11 k	4.37	-8.527354	-4.5	40.02	-18.04
23	A16	7.43	-8.509283	-7.5	59.91	-17.63
24	A17	7.37	-8.497904	-7.2	47.57	ND
25	A14	7.74	-8.497536	-7.1	51.87	-16.17
26	B27	7.85	-8.416398	-7.3	56.36	-17.62
27	10a	4.79	-8.404483	-7.4	56	-17.51
28	11i	4.28	-8.376978	-5.4	44.83	-18.15
29	B25	7.25	-8.366087	**-8.7**	58.6	-21.6
30	10e	4.25	-8.366087	**-8.7**	**60**	-21.8
31	11 t	5.04	-8.309559	-4.5	40	ND
32	11p	4.56	-8.237691	-5.8	39.37	-11.19
33	18	7.83	-8.195755	-6.9	51.11	-19.33
34	11f	4.72	-8.194806	-4.7	44.48	ND
35	dbc-amp	5.67	-8.177967	**-8.4**	**64.44**	-19.67
36	11r	4.23	-8.096362	-4.5	48.35	-15.37
37	11 g	4.73	-8.094617	-4.8	44.78	-18.91
38	A5	7.55	-8.016981	-7.6	50.77	-14.78
39	11n	5.04	-8.002397	-5.1	53.35	-11.13
40	11 m	NA	-8.002397	-6.7	49.27	-11.13
41	11b	4.49	-7.910209	-5	41.77	-18.51
42	B28	7.8	-7.861473	-7.2	59.97	**-24.45**
43	11c	4.06	-7.86083	-4.5	46.85	-17.83
44	11q	6.39	-7.821584	-4.7	48.09	-16.1
45	17	7.91	-7.810211	-6.6	**63.09**	-18.35
46	B24	7.96	-7.760382	-7.9	**62.43**	-19.28
47	8a	4.92	-7.682957	-7.5	55.05	-17.4
48	11 s	4.98	-7.644275	-5.3	42.3	-16.93
49	A11	7.49	-7.604273	**-8.6**	**62**	**-23.45**
50	A15	7.68	-7.576113	-7.5	**61.77**	-17.13
51	B26	7.19	-7.290902	-7.7	50.41	-18.17
52	A13	8.04	-7.28357	-5	60.21	-14.29
53	A1	7.36	-6.714446	-7	50.72	-15.67
54	20b	6.95	ND	**-8.8**	**62.58**	-18.29
55	20c	8.09	ND	**-8.7**	**63.83**	-18.32
56	Ramelotinine	8.5	ND	**-8.3**	45.36	-16.17
57	16	6.5	ND	-7.8	60.81	**-22.52**
58	2a	6	ND	-9	56	-19.08
59	4	7.06	ND	-7.8	57	-20.4
60	5-Methoxy	3.82	ND	-6.7	45.51	-20.56
61	11	2.68	ND	**-8.2**	51.08	-16.31
62	12c	7.93	ND	-7.8	55.42	**-23.75**
63	20a	6	ND	-7.9	59.03	**-24.08**
64	A9	7.27	ND	-4.3	43.31	**-22.65**
65	A10	7.96	ND	-4	54.87	-19.04
66	19	5.12	ND	-7.1	51.87	-16.06
67	20	6.23	ND	**-8.3**	57.53	**-22.06**
68	Melotinine	6.79	ND	-7.1	50.47	-17.51
69	N-Acetyl	5.15	ND	-7.2	44.88	-19.38
70	Agomalotinine	8.67	ND	-4.9	49.75	-14.74
71	SAM	8.04	ND	-6	45.21	-17.46
72	A8	7.85	ND	-6.8	50.18	-15.06
73	A12	7.29	ND	-7.1	37.66	ND

Highlighted ones are the best docking hits according to the corresponding affinities and scores.

ND: Not Docked.

The performance of FRED with respect to ranking the inhibitors in the list was poor. FRED ranked inhibitor B22 on the 11^th^ place whereas all the remaining routines ranked B22 in 1^st^ place. FRED docked fifty three inhibitors out of seventy three provided inhibitors which is a low number as compared to other routines. Kellenberger et al., concluded in their work that FRED had problems with small, polar and buried ligands [37]. FlexX docked sixty eight inhibitors out of seventy three. The most severe restriction of FlexX is that it treats receptor as a rigid entity. Leach in 1994 reported an approach handling receptor flexibility [40].

In the light of the above analysis, the B22ASMT docked pose generated by AutoDock/Vina produced the best results. It forms hydrogen bonds, hydrophobic and ionic interactions with the important residues of the binding pocket of ASMT thus stabilizing the structure of target receptor. The dock pose with least binding energy has the highest affinity and hence is the best docked conformation.

## Conclusion

Docking and scoring have evolved significantly over the past years. It has become a valuable tool in drug discovery process. Our goal of this study was to explore the feasibility of four different docking approaches: (AutoDock/Vina, GOLD, FRED and FlexX) for our target ASMT and to find out the lead compound. We compared the predictive power of each docking and scoring function. Our results suggest that all docking programs studied here do a reasonable job in docking and should aid significantly the drug discovery process. However, AutoDock/Vina consistently outperformed as compared to other programs and was found to be relatively more useful in blind docking pose prediction. Moreover, analysis of the docked ligands with the protein brought into focus some important interactions operating at the molecular level. The results of the ligand docking showed that the binding pocket involves the amino acid residues Ser213, Ser98, Val97, Thr100, Val211, Ser227, Arg210, Arg280, Phe212, Leu198, Ile198, Ser104, Thr195, Leu160, Tyr327, Tyr108, Trp117, Leu326, Phe29, Phe19, Gln334, Asn330, Ala159, Lys107, Met105. The important hydrogen bond forming amino acid residues was Arg280, Thr100, Tyr108, Asn330, Trp117 and Tyr327. In conclusion we have discovered a highly potent lead compound which will be useful for the design of novel less toxic and highly efficient drug for the treatment of bipolar disorders and PPTs.

## Competing interests

The author declares that they have no competing interests.

## Authors’ contributions

SSA has revised the draft critically for important intellectual content as well as has given final approval of the version to be published. SWA carried out computational work and has been involved in drafting the manuscript. Both authors read and approved the final manuscript.

## Supplementary Material

Additional file 1 2D view of melatoninergic inhibitors. Click here for file
